# Challenges and opportunities of artificial intelligence implementation within sports science and sports medicine teams

**DOI:** 10.3389/fspor.2024.1332427

**Published:** 2024-05-20

**Authors:** Mitchell Naughton, Paul M. Salmon, Heidi R. Compton, Scott McLean

**Affiliations:** ^1^ School of Biomedical Science and Pharmacy, University of Newcastle, Callaghan, NSW, Australia; ^2^ Applied Sports Science and Exercise Testing Laboratory, University of Newcastle, Ourimbah, NSW, Australia; ^3^ Centre for Human Factors and Sociotechnical Systems, University of the Sunshine Coast, Sippy Downs, QLD, Australia

**Keywords:** automation, artificial intelligence, multidisciplinary team, teamwork, human-autonomy teaming

## Abstract

The rapid progress in the development of automation and artificial intelligence (AI) technologies, such as ChatGPT, represents a step-wise change in human's interactions with technology as part of a broader complex, sociotechnical system. Based on historical parallels to the present moment, such changes are likely to bring forth structural shifts to the nature of work, where near and future technologies will occupy key roles as workers or assistants in sports science and sports medicine multidisciplinary teams (MDTs). This envisioned future may bring enormous benefits, as well as a raft of potential challenges. These challenges include the potential to remove many human roles and allocate them to semi- or fully-autonomous AI. Removing such roles and tasks from humans will make many current jobs and careers untenable, leaving a set of difficult and unrewarding tasks for the humans that remain. Paradoxically, replacing humans with technology increases system complexity and makes them more prone to failure. The automation and AI boom also brings substantial opportunities. Among them are automated sentiment analysis and Digital Twin technologies which may reveal novel insights into athlete health and wellbeing and team tactical patterns, respectively. However, without due consideration of the interactions between humans and technology in the broader system of sport, adverse impacts are likely to be felt. Human and AI teamwork may require new ways of thinking.

## Introduction

The development in artificial intelligence (AI) systems represent a step-wise change in our interactions with technology in broader society which has implications for sport. Key among recent innovations and at the forefront of this are the recent conversational large language model (LLM)-based chatbot AI's, such as ChatGPT, Microsoft's Copilot AI, and Google's Bard/Gemini. These LLMs are trained on a large corpus (e.g., −570 GB) of textual data (e.g., books, journals, the internet), and allow human users to enter prompts or questions and engage the AI into responding using human-like expressions. The technical process of LLMs relies on attempting to continually predict the next word in sentence structures (1), whilst there are suggestions that ChatGPT may exhibit general intelligence properties which could be viewed as an early (but incomplete) artificial general intelligence (AGI) (2). For these LLMs, we are at or approaching the peak of the Gartner technology “hype cycle”—the general path a technology takes over time, in terms of expectations or visibility of the value of the technology (3). Other automation and AI applications which have relevance to sport include computer vision technology to automatically tag sporting events (4), and tools to produce generative images (e.g., Sora, DALL·E, MidJourney), and data analysis code (e.g., GitHub's Copilot) (5, 6).

Perhaps unsurprisingly, given the capability and ease to draw upon such a large substrate of information, LLMs have begun to be used to assist or replace human work. For example, to co-author academic manuscripts (1), and to pass standardised testing such as the United States Medical Licensing Examination theory section (7). Further, incorporating AI technologies is regarded as a priority of several national governmental policies in areas of national significance, such as defence and healthcare (8, 9). A custom sports medicine themed GPT model called VICTOR has also recently been published (https://chat.openai.com/g/g-h1Es6tIdy-victor-the-evidence-based-sports-medicine-expert). Future generations of AI are likely to develop further complexity and sophistication, such that they may become important assistants and workers, or even replace roles (such as analysts) within sports science and sports medicine multi-disciplinary teams (MDTs). Sports science and sports medicine MDTs typically include different roles with varying specialties across performance and medicine/rehabilitation focused roles such as sports scientist, strength and conditioning coach, performance analyst, physiotherapist, rehabilitation coach, and doctor. MDTs may also include technical/tactical coaches, surgeons, nutritionists, massage therapists, sport psychologists, biomechanists, and other practitioners (10, 11). The exact makeup of this team will vary according to the environment (e.g., professional vs. semi-professional vs. amateur), the resources available, and the sport (e.g., team vs. individual sport). MDT members service various functions which are ultimately aimed at improving the performance and maintaining or improving the health of the athlete(s) they work whilst working in an integrated manner (10). The design and implementation of AI in these sport settings should not be considered in isolation from those who are going to implement it, work with it, or be impacted by its use (12). This envisioned future brings with it challenges and opportunities, included in previously established “ironies of automation” (13) and more recently “ironies of artificial intelligence” (14) which raise questions around the current trajectory.

Given the interactions between technical (or technological) innovations and humans in society, these developments can be understood through a complex sociotechnical system lens (15). Sociotechnical system theory (16) is an approach to understanding and optimising work systems, developed to optimise technology insertion in work systems which include technical and human (or societal) elements. Rather than focusing on the human operator or the technology under investigation in isolation, this approach emphasises the need to optimisate the performance of both (termed “joint optimisation”) (16), with a large body of work supporting the benefits of adopting a sociotechnical system approach (17). Using recent tangible examples and drawing on historical parallels, the goal of this commentary is to provide our perspective on the challenges and opportunities that are likely to emerge within the sports science and sports medicine MDTs during the forthcoming artificial intelligence and automation revolution.

## Challenges

The rapid development and implementation of AI is likely to bring with it structural shifts in how certain tasks are completed in sports science, challenging the current paradigm of work within these fields. An example of this is the First and Second Industrial Revolutions (−1,733–1,913) (18), where, there was a shift from an agriculture-based society to an industrialised one, fundamentally changing the nature of work and the labour market due to technological advances (19). In dominant industries, certain jobs were eliminated, and some career paths declined, ultimately leading to a period of wage stagnation. A specific example here is the development of the automobile and the internal combustion engine which devastated the horse-drawn carriage and steam engine associated jobs, respectively.

Parallels to the current AI boom suggest that jobs and careers within the MDT which rely on tasks that can be or are likely to be fully automated through AI are most at risk. For example, automated computer vision with event detection algorithms will likely remove the necessity of performance analysts to manually code events from video in the future. Further, the need for athletes to wear global/local positioning system (GPS/LPS) devices for external load quantification in team sports (e.g., measuring player velocity, acceleration/deceleration etc.) may be removed with the adoption of vision-based tracking (20). Moreover, the ability to prompt an AI (such as GitHub's Copilot) to accurately analyse data may remove the necessity for sports scientists to develop code for data wrangling, analysis, and visualisation solutions. Using such AI would remove some of the core tasks sports science currently undertake in many team sports (21). However, by removing human tasks through automation and AI, the set of left-over tasks are often more difficult for the human operator leading to performance decrements and skill degradation-related issues when the human operator is required to take over (13). Humans may also lack the skills necessary to undertake the remaining tasks. Further, shifting the human out of the day-to-day loop in performance-related tasks and instead onto a technology supervisory loop role which can create issues, such as a loss of situational awareness and slower response times, which have been catastrophic in other domains (22, 23). This is important as there will likely always be some required level of human involvement with automated systems such as AI, and completely automating certain higher-risk systems may not be acceptable to broader society (e.g., healthcare, autonomous transport) (24). In the context of sport science, AI (at this stage) cannot replicate many of the tasks requiring human intervention, for example interpreting and making decisions from data and communicating with stakeholders and athletes (25). Therefore, teamwork models which identify the optimal working interactions between the human operators and AI as part of the sociotechnical system of the MDT need to be developed (26).

The increased utilisation of AI naturally creates a challenge in that the jobs and careers that are likely to be lost in the future are tangible and presently occupied by humans, while the jobs that will be created by increasing automation and AI are likely unimaginable in the present day. Other potential issues which have yet to be adequately rectified include a high risk of bias, data governance and other ethical issues (e.g., prevention of harm, fairness, privacy, transparency and explainability, accountability etc.), malicious use (e.g., development of new performance enhancing drugs), and a lack of established performance, regulation, and safety in real-world settings where teams of workers and AI work co-operatively (8). It is also likely to change the requirements of education and training.

Outside of the expected challenges, there are likely to be unintended consequences of the AI expansion. One such example in sport is a potential for widening inequality between the teams and organisations with the financial resources to implement more powerful AI than their competitors. This could allow these teams to identify and exploit previously unidentifiable advantages which their less resource laden rivals could not, further exacerbating already existing inequalities in a “winner takes all” scenario (27). This could also, in turn, act as a recruitment draw, or even optimise the accurate detection of talent leading to better quality players and staff. This can be considered an example of the Matthew effect—that advantage begets further advantage (28).

The unbounded development of AI in sport could lead to situations of ethical concern, including AI that could be used to identify and develop undetectable performance enhancing drugs or AI that could uncover winning betting strategies by simulating tactical match-ups and outcomes in near real time. Beyond these foreseeable examples of malicious use there will likely be a host of emergent properties that create new and unforeseen risks. How the transition to an AI future is managed, which institutions manage or regulate it, what controls are put in place, and who ultimately benefits are currently open questions that are critical to consider. Optimising both the human and technological aspects of this complex sociotechnical system appear important to ensure that the conditions for success within the MDT are met and that a “race to the bottom” which leaves people in its wake does not occur. Alarmingly, history tells us that this “joint-optimisation” (29) is not often attempted, let alone achieved (30).

## Opportunities

Whilst the potential challenges of the increased use of AI are considerable, so too are the potential opportunities. As noted briefly above, there were challenges associated with technological advancements during the Industrial Revolutions, however, these periods created rapid increases in the productivity and quality of life for the average citizen as well as increased gross national products of the industrialised countries.

Overall, if joint optimisation of humans and AI is achieved and appropriate controls are in place, it is likely that performance in all sporting domains will be enhanced through the adoption of AI. In a broad sense, increasing implementation of AI in sport may deliver deeper knowledge and optimization to areas such as sports performance, injury prevention, talent identification, nutrition. and training optimisation. When considering sports science and sports medicine MDTs, a potential benefit of the development and use of the Multidisciplinary Human-Autonomy Team (MD-HAT) is the likely similar increase in worker productivity and efficiency that could occur. For example, it is foreseeable that medical staff would be able to query an AI chatbot regarding the symptoms that an athlete is currently experiencing while integrating imaging (e.g., ultrasound, MRI) data and receiving information on a potential differential diagnosis in return (31). Whilst this is unlikely to replace the final decision making of a sports medical practitioner, it can provide complementary information which can be incorporated in the decision-making process. Critical design considerations here are transparency, explainability, and distributed situation awareness—ensuring that human team members can understand what the AI is aware of, what information it is using, and how it arrives at its recommendation.

Another foreseeable opportunity is the removal of manually laborious tasks such as recording and entry of athlete data from the human management of the MDT entirely. During the introduction, this may increase the human workload as there will be a transition period whereby a human MDT worker will need to manually check the performance and accuracy of the AI, but eventually this could allow staff within the MDT more time to work more directly with athletes and coaches. This time could be used to develop rapport (identified of high perceived value to both coach and MDT practitioner) (32), and resolve one of key barriers (in lack of time) to implementing injury prevention programmes (33).

Further potential opportunities for automation and AI include Digital Twin models of health and performance (34) Digital Twins are virtual counterparts of physical objects or systems that are dynamically updated over time using data collected in the real world (35). For example, a Digital Twin of an athlete could be created using data collected from wearable sensors, biometric devices, motion capture, video analysis, and other sources of athlete information (e.g., health and training records). This Digital Twin can then be used to simulate the athletes performance under different conditions, such as a new training regime, nutrition plan, recovery intervention, and environmental factors (34). Digital Twins of teams could be simulated repeatedly under different tactical systems to assess their suitability. Recruitment staff could use Digital Twins of coaches to understand how different coaches tactical formations and instruction would influence the behaviours of a current squad of players.

Sentiment analysis is analysing athlete spoken or written language for different cues which may provide an indication of underlying emotional states (36). Automating sentiment analysis of athlete speech, for example, may allow sports science practitioners to question athletes regarding subjective health and wellbeing (such as their current fatigue or mood state) and analyse the underlying sentiment behind the athletes chosen response(s). This may assist in understanding athlete subjective wellbeing by rectifying one of the key limitations of using subjective monitoring, namely misleading responses given by athletes (37). Accurate representations of an athletes cognitive/perceptual state would allow for further analysis of the relationships between subjective and objective (e.g., neuromuscular, cardioautonomic) indicators of recovery and fatigue which can, in turn, influence performance improvement and injury risk reduction (38).

Whilst these are foreseeable applications which use current or near-term automation and AI advancements, there are many such developments which have not been imagined yet. Not all these scenarios involve well-intentioned, ethical, or non-financially incentivized actors. A future in which these AI develop into an AGI which applies itself to sport is possible (39).

## Systems thinking analysis of AI insertion in sport

**Figure 1:**
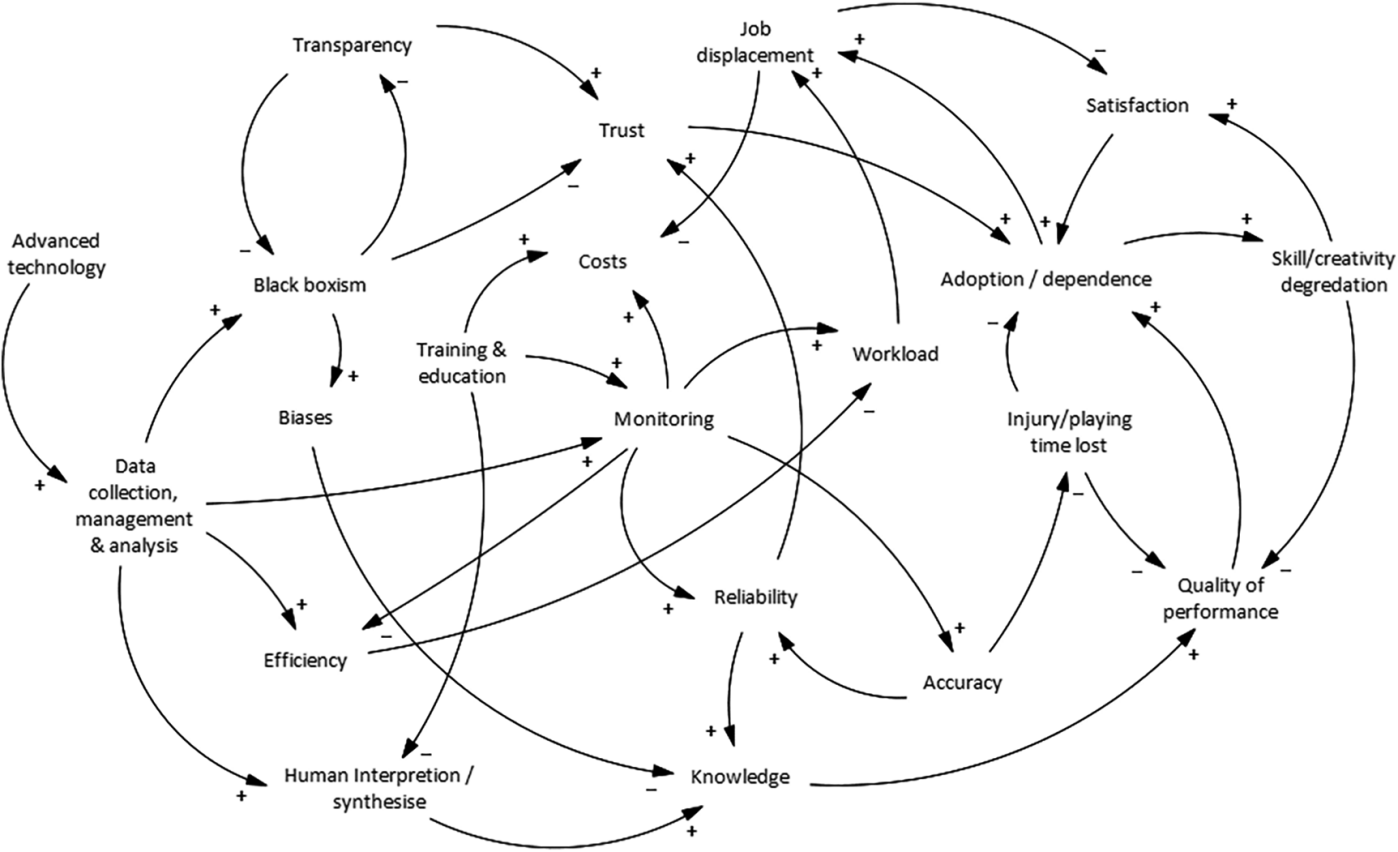
Causal loop diagram (CLD) describing the reinforcing [positive feedback (+)] and balancing [negative feedback (−)] loops involved in the implementation of advanced artificial intelligence (AI) and automation technologies to sports.

The inherent complexities, challenges, and opportunities associated with AI and automation technology implementation to sport are summarised through a Causal Loop Diagram (CLD) (40) in Figure 1. The CLD depicts the feedback loops that influence behaviour in a given system. Within the CLD, positive loops identify how the effect of a change in one variable leads to an increase in the same variable, which in turn leads to further increases in that variable. For example, an increase in the reliability of an AI will lead to an increase in human users' trust in the AI (41) (Figure 1). Whereas, in a negative loop, a change in a variable lead to an opposite change in another variable, which in turn leads to a counterbalancing effect that reduces the initial change. For example, an increase in skill/creativity degradation leads to a decrease in job satisfaction (42) (Figure 1).

The CLD demonstrates several reinforcing and balancing loops that will likely be involved in the insertion of AI and automation technologies in MDTs. This illustrates the complex nature of sports science and sports medicine MDTs and the multiple variables and pathways which are necessary to consider when assessing the likely impact of AI. Lastly, from the CLD, it appears that even the most optimal application of AI in sport may not bring immediately identifiable benefits to MDTs without consideration of joint-optimisation of both the human and technological elements.

Given the potential challenges and opportunities to the MDT with the implementation of AI and automation in sport, successful integration requires a series of appropriate and well considered steps. Makarius et al. (43) provides a sociotechnical framework with which to integrate artificial intelligence into the work environment. This encompasses a series of four sequential phases from the initial employee anticipation phase, to the AI-employee encountering phase, to the symbiotic metamorphosis phase, and finally the sociotechnical capital phase (43). Ultimately, successfully managing the integration leads to the final sociotechnical capital stage where the AI is integrated in the human-AI work environment. The associated competitive advantages of doing this to the organisation are therefore realised. In the context of sports science and sports medicine MDTs, this would be the successful coworking of human and AI within the MDT to achieve the functions of improved performance and the maintenance or improvement of athlete(s) health.

## Discussion

“*Machine intelligence is the last invention that humanity will ever need to make.*”—Nick Bostrom

The future seems increasingly driven by the growing proliferation and adoption of AI, and the influence of this shift will inevitably shape the environment and work practices of sports science and sports medicine MDTs. Importantly, as noted by Bainbridge (13) in the 1980's, there is a key irony at the heart of the shift towards increasing automation in that the more advanced a control system, the more critical the contribution of the human operator. This is true for the current iteration of AI, such as ChatGPT, where the output is conditional upon the content and quality of the prompts given [and interpretation of the results by the interacting human(s)] (2). This is likely to be true in future iterations which require constant (or more frequent) human-AI collaboration and teamwork.

Crucially, there are likely to be challenges, and unintended consequences and issues which arise with the broad-based application of AI in the sports science and sports medicine MDT. Indeed, history is littered with examples of new and advanced technologies which behave unexpectedly or lead to unintended consequences when introduced (e.g., the printing press, splitting of the atom, or social media). Without due consideration of the interaction between humans and technology in a sociotechnical system, adverse performance and negative impacts to the individuals involved will be felt. However, whilst the likely challenges are considerable and there will be much angst about how the nature of work and careers will change in sporting contexts, the potential opportunities to the MDT associated with increasing automation and AI are just as substantive and should be recognised.

In speculating, AI and automation has the potential to reduce labor costs and improve efficiency, which may result in fewer roles and humans to staff those roles within MDTs. This potentially introduces ethical issues which further expand to include data privacy, security, inherent biases, and equity as AI systems may amplify pre-existing issues. To make AI trustworthy for shared ethical decision making collaboration between all relevant stakeholders is required (44). The proliferation of AI may also change the nature of sports science and sports medicine education as a skillset which was previously developed through education is no longer relevant. Understanding the risks associated with AI and it's integration can and should be undertaken prospectively so that adequate controls can be developed and implemented (24). What the benefits are of increasing automation and AI, and who ultimately benefits, are yet to be fully realised. We hope that this commentary has given researchers and practitioners food for thought, and that our discipline engages in the work required to ensure that sport systems benefit from safe, ethical, and usable AI.
